# Neutrophil-to-Lymphocyte Ratio Facilitates Identification of Obstructive Sleep Apnea in Patients with Type B Aortic Dissection

**DOI:** 10.1155/2021/8492468

**Published:** 2021-11-30

**Authors:** Dandan Jiang, Qu Chen, Weiming Su, Dinghui Wu

**Affiliations:** ^1^Department of Pulmonary Medicine, Xinglin Branch of The First Affiliated Hospital of Xiamen University, Xiamen, Fujian Province, China; ^2^Department of Cardiovascular Surgery, The First Affiliated Hospital of Xiamen University, Xiamen, Fujian Province, China

## Abstract

**Purpose:**

To determine whether the neutrophil-to-lymphocyte ratio (NLR) aids in the detection of obstructive sleep apnea (OSA) in patients with type B aortic dissection (TBAD).

**Methods:**

324 patients with TBAD or type B aortic intramural hematoma (TB-AIMH) underwent an overnight sleep study. We divided the eligible 256 studied subjects into three groups: group A (*n* = 109, TBAD patients with OSA), group B (*n* = 68, TB-AIMH patients with OSA), and group C (*n* = 79, TBAD patients without OSA). Baseline characteristics, biochemical and sleep parameters, and STOP-Bang questionnaire scores were collected. To assess the predictive efficacy of potential variables, multivariate logistic regression analysis and receiver operating characteristic (ROC) curves were used.

**Results:**

The study found that about 58% of TBAD patients and 54% of TB-AIMH patients had OSA, a majority of whom had moderate to severe OSA (95.41% and 89.71%, respectively). In the comparison of sleep parameters between patients with TBAD and TB-AIMH, no other than apnea and hypopnea index (AHI) made a significant difference. The multivariate logistic regression analysis showed that neutrophil-to-lymphocyte ratio (NLR) (odds ratio (OR): 3.614, 95% confidence interval (CI): 2.273–5.748, and *P* < 0.05) and STOP-Bang scores (OR: 1.97, 95% CI: 1.34–2.90, and *P* < 0.05) were both independent predictors for OSA in patients with TBAD. ROC curves showed NLR had higher sensitivity (65% versus 59%) and specificity (86% versus 57%) for OSA than the STOP-Bang questionnaire. Furthermore, NLR was positively correlated with AHI through the Spearman test (*r* = 0.398 and *P* < 0.05).

**Conclusion:**

NLR was an independent predictor of OSA in TBAD patients with higher sensitivity and specificity than the STOP-Bang questionnaire, and it was positively associated with AHI. NLR may aid in the diagnosis and risk stratification of OSA in TBAD patients.

## 1. Introduction

Obstructive sleep apnea (OSA) is a common clinical condition characterized by repetitive episodes of apnea or hypopnea with recurrent collapse of the upper airway tract during sleep, resulting in shortened sleep duration and poor sleep quality due to intermittent hypoxia, sleep fragmentation, oxidative stress, and other unclear complex pathophysiological mechanisms [1]. OSA is associated with various detrimental complications, including cognitive impairment, cardiovascular diseases, pulmonary diseases, endocrine dysfunction, and neuropsychiatric problems [2–4], among which cardiovascular diseases are of primary concern due to their higher incidence, acute onset, and poor prognosis. In recent years, the increasing prevalence and comorbidity of OSA and aortic dissection have captured great attention [5–7].

Aortic dissection (AD) is a life-threatening disease and derives from a tear in the intimal layer of the aorta resulting in the entry of blood into the space between the intima and the media, which is usually accompanied by the classic symptom of “severe tear pain.” As studies indicate, AD patients who do not receive optimal medical treatment and instant rescue have a mortality rate of 50–68% during the first 48 hours after onset, with a mean mortality of up to 1% per hour and up to 90% within 3 months of occurrence [8]. Acute aortic syndrome is described as a series of life-threatening aortic diseases which include aortic intramural hematoma (AIMH) and penetrating atherosclerosis ulcers besides AD. Both AD and AIMH could be categorized into two types according to the classical Stanford system: type A and type B [9]. Lesions involving the ascending aorta are classified as type A, whereas those involving only the descending aorta are classified as type B. Empirically, the former type usually needs an immediate operation, and the latter could survive with optimal medication treatment.

Recently, an increasing number of observational studies have demonstrated a high prevalence of previously undiagnosed OSA in patients with AD [10–12]. This is because they are usually unaware of it and disregard signs such as excessive daytime sleepiness, fatigue, snoring, nocturia, or resistant hypertension. OSA-induced intermittent nocturnal hypoxia and sleep fragmentation create a cascade of hemodymic and neuroendocrine changes [13], which precipitate the onset and progression of AD [14]. If the pathogenetic alterations are not addressed promptly and efficiently, they will persist, resulting in worse complications and a poor prognosis for AD [6]. Early and precise diagnosis of OSA in AD patients would aid in prioritizing patients for sleep study and therapy, which would improve the prognosis of patients with comorbidity.

While overnight polysomnography (PSG) is the gold standard for OSA diagnosis, it is costly, time-consuming, and difficult to operate on severely ill patients. Numerous questionnaires are currently used to screen for OSA, one of which is the Stop-Bang questionnaire [15]. It is widely used for its simplicity and high sensitivity, whereas some patients without typical symptoms are likely to be overlooked and an imprecise estimate of self-reported symptoms reduces its predictive accuracy for OSA. Therefore, it is critical to develop a readily accessible but more trustworthy screening tool. Neutrophil-to-lymphocyte ratio (NLR) is a novel inflammatory biomarker that has been proven to be not only associated with OSA but also to indicate its severity [16]. Given this, it is worthwhile to investigate if NLR could be utilized to screen for OSA in AD patients and how accurate it is compared to the STOP-Bang questionnaire. To ensure the study's reliability, we excluded patients with type A aortic dissection (TAAD) who required emergency operations because operative trauma or complications could have affected the normal sleep cycle. The purpose of this study was to ascertain the prevalence of OSA in patients with type B aortic dissection (TBAD), as well as the predictive value of NLR in TBAD patients for OSA.

## 2. Materials and Methods

### 2.1. Patient Selection

354 patients with AD and 269 patients with AIMH were admitted to the Department of Cardiovascular Surgery, First Affiliated Hospital of Xiamen University, from January 2017 to January 2021. The exclusion criteria are as follows: (1) patients whose age were not between 18 and 80; (2) patients with TAAD or type A aortic intramural hematoma; (3) patients with aortic diseases and thoracic operation previously; (4) patients receiving sleep-disordered treatment, such as continuous positive airway pressure or medication; (5) patients with chronic obstructive pulmonary disease; (6) patients who did not consent to be enrolled in this study; (7) patients with infectious disease or systemic inflammatory disease, recent neoplasm history, autoimmune disease, acute renal failure, acute limb ischemia, and unstable hemodynamics; and (8) other circumstances which would disrupt normal sleep cycles. Finally, we enrolled 324 eligible patients in the study. The STOP-Bang questionnaire and overnight sleep study with PSG were administered in the sleep center of this hospital. According to the PSG results, 4 patients with TBAD and 6 patients with type B aortic intramural hematoma (TB-AIMH) were excluded from this study because the recording duration available for the sleep study was shorter than 3 hours. We divided the remaining 256 studied subjects into three groups: group A (*n* = 109, TBAD patients with OSA), group B (*n* = 68, TB-AIMH patients with OSA), and group C (*n* = 79, TBAD patients without OSA), apart from the fifty-eight TB-AIMH patients without OSA.

**Figure 1:**
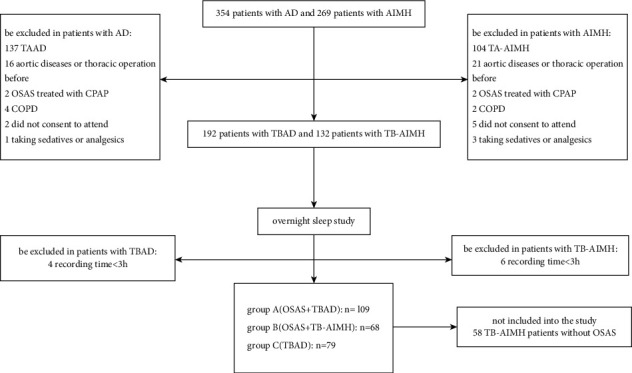
Study flowchart of patients' selection process.

The flowchart of patient selection is shown in Figure 1. This study complied with the Declaration of Helsinki, and the study protocol was approved by the Institutional Ethical Committee of the First Affiliated Hospital of Xiamen University (Xiamen, China). All participants in this study provided written informed consent.

### 2.2. STOP-Bang Questionnaire and Overnight Polysomnography

The STOP-Bang questionnaire was performed before the PSG test and proceeded with the instruction of the specific physician (WS), who was blind to the PSG results. The detailed items of the STOP-Bang questionnaire are listed in Table 1. It has eight dichotomous (yes/no) questions about the clinical features of sleep apnea (snoring, tiredness, observed apnea, high blood pressure, BMI, age, neck circumference, and male gender). For each question, answering “yes” scores 1, a “no” response scores 0, and the total score ranges from 0 to 8.

All patients underwent sleep evaluation for one night with the use of a SOMNOcheck 2 R&K instrument (Weinmann, Hamburg, Germany). PSG consisted of an electroencephalogram (EEG, C4/A1, and C3/A2), right and left electrooculograms (EOGs), a submental electromyogram (EMG), an electrocardiogram (ECG), chest and abdominal movement, airflow detection (detected by a nasal-oral thermocouple), and an oxygen saturation (SpO_2_) evaluation by finger pulse oximetry.

### 2.3. Definition of Sleep Events and Diagnosis of Related Diseases

The results were analyzed by the sleep physician (DJ), who was blind to the STOP-Bang questionnaire scores of the patients, according to the recommendations of the American Academy of Sleep Medicine Scoring Rules [17]. Score an apnea when all the following criteria are met: (1) there is a drop in the peak thermal sensor excursion by ≥90% of baseline and (2) the duration of the event lasts at least 10 seconds. Hypopnea definition requires a 30% or greater drop in flow for 10 seconds or longer associated with ≥3% oxygen desaturation or an arousal. An event of microarousal was defined as an abrupt shift in the EEG frequency, including alpha, theta, and/or frequencies >16 Hz (but not spindles) that lasted at least 3 seconds, with at least 10 seconds of stable sleep preceding the change. The oxygen desaturation index (ODI) in this study was defined as the total events per hour when at least 3% decreased from the baseline oxygen saturation monitored by the finger pulse oximetry. Likewise, the apnea and hypopnea index (AHI) and microarousal index (MAI) are defined by the number of related events per hour of recording. In addition, total sleep time (TST), the percentage of sleep efficiency (SE), the lowest oxygen desaturation during recording (LSpO_2_), and the percentage of time spent at oxygen desaturation <90% (*T* < 90%) were also collected. The diagnosis of OSA was defined when AHI ≥5 events per hour based on an overnight sleep study with PSG. The severity of OSA was determined by AHI: mild type (AH ≥ 5 events/h), moderate type (AHI ≥ 15 events/h), and severe type (AHI ≥ 30 events/h).

Diagnosis of AD or AIMH was based on morphological findings of enhanced computer tomography with or without typical clinical symptoms, including the acute onset of severe chest pain and back pain radiating to the neck or shoulders. The diagnosis of AD and AIMH, as well as Stanford's type of every patient, was determined by the cardiovascular surgeon (QC) who was blind to the PSG results.

### 2.4. Hematological Parameters

For all patients in the three groups, blood samples were obtained on admission and were analyzed within 1 hour after venepuncture to avoid alterations in parameters due to prolonged storage at room temperature. Biochemical parameters, including white blood cell count (WBC), neutrophil percentage, lymphocyte percentage, platelet count (PLT), mean platelet volume (MPV), CRP, and D-dimer, were collected. The NLR, platelet to lymphocyte ratio (PLR), and mean platelet volume to platelet ratio (MPV/PLT) were all calculated from blood test data.

### 2.5. Statistical Analysis

Quantitative variables were presented as mean ± standard deviations if normally distributed, and median (25^th^ and 75th percentile) if abnormally distributed. Qualitative variables were expressed as counts and percentages. Normality was analyzed using the Shapiro–Wilk test. In three-group comparisons of continuous data, normally distributed parameters were compared using the one-way ANOVA test, whereas non-normally distributed parameters were compared using the Kruskal–Wallis test, with Bonferroni adjustment for post hoc analysis. Categorical variables were compared by Pearson's chi-square test. Significant variables were selected if they were related with OSA at the *P* < 0.1 level in univariate logistic regression analysis and then entered into a multivariate logistic regression model to discover independent predictors of OSA in TBAD patients. After adjusting for confounders such as BMI, PLR, and MPV/PLT ratio, the NLR and STOP-Bang scale were found to be independently associated with OSA. Receiver operating characteristic (ROC) curve analyses were performed to evaluate the predictive performance of the NLR and STOP-Bang scale. Spearman correlation analysis was used to evaluate the association between NLR and AHI. *P* < 0.05 was considered statistically significant. Data analysis in this study was performed using SPSS Statistics for Windows, Version 22.0 (SPSS Inc., Chicago, Illinois, USA).

## 3. Results

### 3.1. Prevalence of OSA and Demographic Characteristics

**Figure 2:**
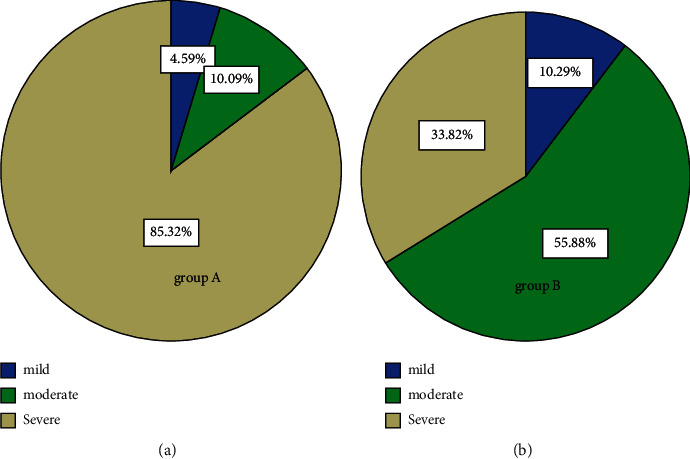
Proportion of mild-to-severe OSA in group A (patients co-occurring OSA and TBAD) and group B (patients co-occurring OSA and TB-AIMH). (a) The proportion of mild-to-severe OSA in patients with TBAD: 4.59% mild OSA, 10.09% moderate OSA, and 85.32% severe OSA. (b) The proportion of mild-to-severe OSA in patients with TB-AIMH: 10.29% mild OSA, 33.82% moderate OSA, and 55.88% severe OSA.

The prevalence of OSA in patients with TBAD reached as high as 58% and 54% of TB-AIMH patients were diagnosed as OSA, no significant difference was observed between two groups. As Figure 2 revealed, the percentage of mild, moderate, and severe OSA was 4.59%, 10.09%, and 85.32% in group A, and 10.29%, 55.88%, and 33.82% in group B, respectively. The overall prevalence of moderate to severe OSA in groups A and B was both considerably high (95.41% and 89.71%, respectively). Severe OSA made up the greatest proportion of patients in group A, while moderate OSA was the most common type in group B.

Baseline characteristics of all the three groups are presented in Table 2. There existed no significant demographic difference in three groups. However, roughly four-fifths of patients in the three groups were all male. The majority of all three groups had smoking and drinking history, hyperglycemia, hypertension, and hyperlipidemia. In terms of symptoms of OSA, daytime sleepiness and snoring during sleep were more prevalent in OSA patients (groups A and B) than in non-OSA patients (group C), although no significant difference was observed between A and B. While hypertension was substantially more prevalent in group A than in group B or C (89.9% in group A, 70.6% in group B, 73.4% in group C, and *P* < 0.05). Likewise, group A scored higher on the STOP-Bang questionnaire than either group B or C.

### 3.2. Hematological Parameters and Sleep Study Results

With regard to hematological parameters (Table 2), NLR was significantly higher in group A (4.36 ± 0.75) than in groups B or C (3.42 ± 0.97 and 3.65 ± 0.65, respectively). TBAD patients with OSA exhibited a higher proportion of neutrophils and lymphocytes compared to both groups B and C (*P* < 0.05). The MPV/PLT ratio was significantly higher in group A than in group B, but no significant difference was found between groups A and C (7.46 ± 2.14, 6.31 ± 1.87, 6.82 ± 1.75 in groups A, B, and C, respectively). There was no significant difference in WBC, PLR, PLT, MPV, CRP, or D-dimer levels between the three groups.

Table 3 summarizes the PSG parameters of patients in three groups. There was no statistically significant difference in TST between the three groups. Patients with aortic disease, whether TBAD or TB-AIMH, exhibited significantly greater AHI, MAI, and ODI, and *T* < 90% and lower SE and LSpO_2_. When comparing groups A and B, only AHI showed a significant difference. There was no significant difference in TST, SE, MAI, ODI, and LSpO_2_ and *T* < 90%.

### 3.3. Variables Related to OSA among TBAD Patients

**Figure 3:**
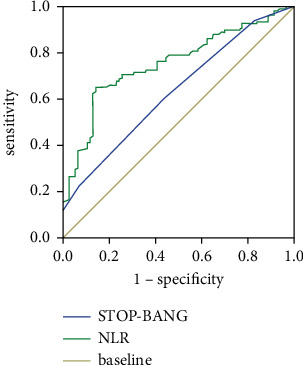
ROC curves of NLR and the STOP-Bang questionnaire for OSA among patients with TBAD (groups A and C).

**Figure 4:**
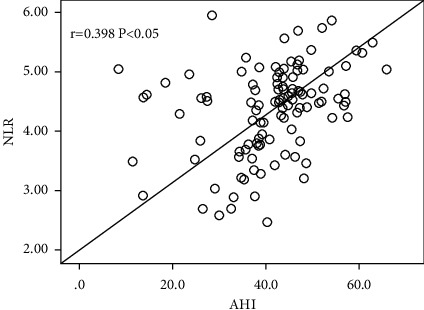
Correlation of AHI and NLR in patients with TBAD patients with OSA (group A). The likelihood of NLR significantly increased along with elevated AHI, namely, the severity of OSA (*r* = 0.398, *P* < 0.05); AHI, apnea, and hypopnea index; NLR, neutrophil-to-lymphocyte ratio; r represents the correlation coefficient.

Logistic regression results are described in Table 4. The NLR, STOP-Bang questionnaire, and MPV/PLT were the variables linked significantly with OSA as indicated by univariate logistic regression (*P* < 0.1). As demonstrated by multivariate regression analysis, only NLR (OR 3.73, 95% CI 2.27–6.15, and *P* < 0.05) and the STOP-Bang questionnaire (OR 1.97, 95% CI 1.34–2.90, *P* < 0.05) were independent predictors for OSA.  Table 5 and Figure 3 illustrate the diagnostic performance of the NLR and STOP-Bang questionnaire for OSA in patients with TBAD. The best cutoff value of NLR was 4.27 with 86.1% specificity and 65.1% sensitivity, and the STOP-Bang questionnaire held 59% sensitivity and 57% specificity at the cutoff value of 3.50. Positive predictive value (PPV) for NLR and the STOP-Bang questionnaire in detecting OSA was 71% and 57.8%, respectively, and negative predictive value (NPV) was 82.2% and 58.1%, respectively. NLR and the STOP-Bang questionnaire had area under ROC curves (AUCs) of 0.75 and 0.63 for OSA, respectively. Furthermore, Spearman correlation analysis showed a positive association between NLR and AHI (*r* = 0.398, *P* < 0.05) in TBAD patients with OSA. As OSA severity grew, Figure 4 demonstrates a progressive increase in NLR.

## 4. Discussion

The primary finding of the study was that NLR and STOP-Bang score were independently associated with OSA in TBAD patients. NLR has a higher sensitivity and specificity for detecting OSA than the STOP-Bang questionnaire. To our knowledge, this is the first time the diagnostic value of the NLR has been explored for the identification of OSA in TBAD patients.

High prevalence of OSA in patients with cardiovascular diseases has been indicated by many observational studies. In the current study, 58 percent of TBAD patients and 54 percent of TB-AIMH patients had OSA, which was comparable to the 66.2 percent among TBAD patients reported by Wang et al. [6]. Zhou revealed an overall 45 percent prevalence of OSA among AD patients [18]. The disparity in prevalence rates may be explained by a small cohort size and a varied distribution of hypertension. Of note, more than 90 percent of patients with TBAD or TB-AIMH had moderate to severe OSA in this investigation, with a higher prevalence of severe OSA in TBAD patients than previously described [6]. As a plausible explanation, a meta-analysis revealed that patients with moderate to severe OSA (AHI ≥ 15 events/h) had a 4.43-fold increased relative risk of developing AD compared to patients with AHI < 15 events/h [18]. With regard to the detailed sleep metrics, no variable other than AHI was found to be significantly different between TBAD patients and TB-AIMH patients. In accordance with the study conducted by Saruhara et al. [12], there were no significant differences in hypoxia markers such as 3% ODI, *T* < 90%, and LSpO_2_ between patients with AD, thoracic aortic aneurysm, and abdominal aortic aneurysm.

The high prevalence of OSA and AD comorbidity attracts increasing interest and fuels the desire to explore the mechanisms underlying OSA and AD. Several mechanisms have been substantiated as possible contributors to comorbidity. Firstly, intermittent hypoxia has been shown to activate the sympathetic nervous system in both animal and human studies [19, 20], and recurrent arousal during sleep will strengthen this effect. As a consequence, end-apneic arterial pressure surges repetitively and tends to be resistant. Furthermore, higher intrathoracic negative pressure was found in OSA patients as a result of increased inspiratory effort against airway occlusion. Suzuki et al. conducted an in vivo study and found that the negative end apneic pressure was −53.6 ± 2.9 cmH_2_O and the peak pressure to be −147.4 cmH_2_O [21]. In comparison, the physiological inspiration pressure is approximately −5 to −8 cmH_2_O in healthy subjects [22]. Changes in intravascular and extravascular pressure produce an obvious elevation in transmural pressure on the aorta and substantial shear stress on its wall, and AD will develop as a consequence [23]. In addition, Bai et al. discovered that the strength of aortic medial fibers was negatively related to serum inflammatory cytokines (IL-6 and TNF-*α*) in OSA patients [24]. This indicates that OSA-related inflammation is likely to impair fiber strength directly and contribute to the development of AD. Moreover, oxidative stress, endothelial dysfunction, and atherosclerosis may be additional risk factors for AD as a joint result of intermittent hypoxia and recurrent arousals [25].

It is critical to identify OSA in the AD population for the following reasons. Firstly, it is reported that OSA was associated with an overall 60% increase in the risk of developing AD, and the risk increased up to 433% between moderate to severe OSA and AD [18]. It suggested that the risk of AD had a positive correlation with the severity of OSA. Actually, OSA may not only contribute to the onset and progression of AD but also impacts long-term outcome in AD patients. Wang et al. studied the effect of OSA on the prognosis of AD in fifty-one OSA patients and forty non-OSA patients. After 36 months of follow-up, there was a statistically significant difference between the study group's all-cause mortality rate of 27.5% (14/51) and the control group's mortality rate of 10.0% (4/40) [26]. In addition, patients with untreated OSA are at increased risk of preoperative anesthesia [27] and postoperative complications and accidents, including difficult intubation, prolonged hospital stay, and increased intensive care unit admissions [28, 29].Furthermore, it is not rare for individuals with undetected OSA and profound hypoxic injury from apnea to be misdiagnosed as cardiac arrest during postoperative period [30]. In addition to having a elevated prevalence of various cardiovascular events, untreated OSA patients have a shorter average life expectancy than the general population [31]. Given the foregoing, it is crucial to develop a readily accessible indicator in order to improve life quality of the patients with AD and OSA.

The STOP-Bang questionnaire includes eight dichotomous (yes/no) questions about the clinical features of sleep apnea (snoring, tiredness, observed apnea, high blood pressure, BMI, age, neck circumference, and male gender). It could be completed quickly and easily (within 1–2 minutes), which was originally used for identifying patients at high risk of OSA in preoperative population [32]. Numerous difficulties have been encountered when the STOP-Bang scale is used in clinical practice. First of all, inadequate specificity results in a higher percentage of false positives, unnecessary referrals to sleep clinics, and a lengthy waiting list for costly and inconvenient PSG testing. Furthermore, its set cutoff point is questionable across various subpopulations. For instance, the threshold for a high risk of OSA may vary depending on the proportion of females in the study sample. Orbea et al. recommended a lower threshold to trigger further testing in women given the nature of its question about gender [33]. Additionally, imprecise scores may usually result from erroneous self-evaluation about the item No. 1–No. 3 in the STOP-Bang questionnaire. On the other hand, several typical features of AD patients, including advanced age, male gender, hypertension, and obesity [34], definitely result in a higher STOP-Bang score, which is likely to be misinterpreted as a higher risk of OSA. Finally, the physician responsible for scoring PSG events is frequently not blind to the STOP-Bang questionnaire score [35], which obviously impairs personal judgment and decreases the reliability of OSA diagnosis. All of these issues are likely to have contributed to prior studies exaggerating the sensitivity of the STOP-Bang questionnaire for OSA.

Among the currently established pathophysiological mechanisms of OSA, chronic systemic inflammation has been verified to induce direct aortic medial degeneration [36, 37], which leads to aortic vulnerability and a propensity for AD. In addition, inflammatory markers are more diverse, stable, and easier to monitor than hemodynamic markers, both of which are implicated in the etiology of AD. Previous studies have established several inflammatory markers for detecting OSA, but no one has attempted this in AD patients to our knowledge. Mutlu et al. demonstrated that high serum YKL-40 levels correlated with AHI and might serve as a predictive biomarker for OSA [38]. Sozer et al. also claimed that pentraxin-3, an inflammatory biomarker, could be a critical predictor of OSA and its severity [39]. NLR is a widely accepted and reliable indicator for systemic inflammation implying a higher inflammatory burden, which reflects two distinct but complementary immune pathways: nonspecific active inflammation by neutrophils and specific immune regulation by lymphocytes. Consequently, the NLR could be more stable and trustworthy than other inflammatory parameters.

Several previous research studies have shown a strong relationship between NLR and OSA. A meta-analysis comprised eleven eligible studies with 2,259 OSA patients showed that NLR was substantially higher in OSA patients than in controls and was greater in severe OSA patients [16]. Al-Halawani compared the NLR value in OSA patients before and after treatment with mandibular advancement devices. They found that NLR was higher in those with moderate to severe OSA than in those with mild OSA, and in the following subgroup analysis, there was a synchronously significant decline in the NLR and AHI in the group with optimal treatment [40]. There is a paucity of data on the predictive significance of NLR in patients with AD. In present study, we found that NLR was associated with OSA independently and positively correlated with AHI (*r* = 0.398, *P* < 0.05) among TBAD patients. Regarding the correlation of NLR and AHI, Uygur et al. also found that individuals with severe OSA had a substantially greater NLR than those with mild-to-moderate OSA and that NLR was significantly correlated with AHI (*r* = 0.437, *P* < 0.001) [41]. Besides the abovementioned findings, the present study also substantiated that NLR had a sensitivity of 65% and a specificity of 86% for predicting OSA, with a higher diagnostic accuracy than the STOP-Bang questionnaire. This could be because NLR was less likely to be influenced by the varied proportion of women in the study sample or by equivocal judgments regarding symptoms from patients. Our findings suggest that NLR may be a simple, economical, and reliable marker for predicting OSA and its severity in TBAD patients. Further research is anticipated to determine whether NLR could be used to predict therapy efficacy for OSA in TBAD patients.

Limitations in this investigation should be mentioned. First, the inadequate sensitivity of NLR to identify OSA is an obvious drawback in spite of its higher sensitivity and specificity than the STOP-Bang scale. This could be owing to the limited sample size and the higher NLR threshold imposed by the considerable prevalence of severe OSA. We anticipate more research studies with a bigger sample size from diverse ethnic groups, which will improve the stability and reliability of the NLR cutoff value. Second, we could not judge whether NLR was a constant indicator of OSA due to a lack of follow-up regarding subsequent changes in the NLR. Furthermore, we observed hesitancy, insecurity, and irresponsibility on the part of many patients, particularly the elderly, when responding to STOP-Bang questionnaire item No. 1–No. 3. This may result in an imprecise score. With regard to the definition of OSA, it is recommended in ICSD-3rd edition as follows: AHI ≥15 events/h or AHI ≥5 events/h with signs/symptoms or associated medical/psychiatric disorders. In this study, we defined OSA as an AHI ≥5 events/h, and we assumed that TBAD was strongly associated with OSA by default, which might lead to an overestimation of OSA prevalence in the TBAD group. Imprecise scale scores and exaggerated incidence of OSA may reduce the STOP-Bang questionnaire's sensitivity to some extent. However, to our knowledge, this is the first time that the predictive efficacy of NLR for OSA in patients with TBAD has been evaluated. The fundamental strength of the study is that all patients underwent the PSG inlab, which increased the precision of the analyses. According to the conclusion from this study, individuals with TBAD and greater NLR, especially those who snore loudly and experience excessive daytime drowsiness, should have overnight PSG test or portable sleep monitoring conducted.

## 5. Conclusion

OSA was shown to be prevalent in around 58% of patients with TBAD, with the moderate-to-severe type accounting for 95.41% of them. NLR was an independent predictor of OSA in TBAD patients with higher sensitivity and specificity than the STOP-Bang questionnaire, and it was positively associated with AHI. NLR may aid in the diagnosis and risk stratification of OSA in TBAD patients.

## Figures and Tables

**Table 1:** STOP-Bang questionnaire.

Questions	1 score	0 score
(1) Snoring: do you snore loudly (louder than talking or loud enough to be heard through closed doors)?	Yes	No
(2) Tired: do you often feel tired or sleepy during daytime?	Yes	No
(3) Observed: has anyone observed you stop breathing during your sleep?	Yes	No
(4) Blood pressure: do you have or are you being treated for high blood pressure?	Yes	No
(5) BMI: more than 35 kg/m^2^?	Yes	No
(6) Age: over 50 years old?	Yes	No
(7) Neck circumference: >16 inches (40 cm)?	Yes	No
(8) Gender: male gender?	Yes	No

**Table 2:** Comparison of demographic and biochemical characteristics in all the three groups.

Characteristics	Group A (*n* = 109)	Group B (*n* = 68)	Group C (*n* = 79)	*P* value
Age (years)	50.38 ± 11.35	48.72 ± 12.49	47.03 ± 10.57	0.615
BMI (kg/m^2^)	27.88 ± 2.14	27.28 ± 1.83	28.02 ± 2.18	0.133
Neck circumference (cm)	42.19 ± 2.81	43.27 ± 2.69	41.89 ± 2.66	0.760
Waist circumference (cm)	88.61 ± 6.29	87.89 ± 6.13	86.9 ± 5.97	0.167
Male, *n* (%)	88/21 (80.7%)	53/15 (77.9%)	61/18 (77.2%)	0.822
Smoking, *n* (%)	85/24 (78%)	48/20 (70.6%)	60/19 (75.9%)	0.534
Drinking, *n* (%)	89/20 (81.7%)	50/18 (73.5%)	55/24 (69.6%)	0.145
Hypertension, *n* (%)	98/16 (89.9%)	48/20 (70.6%)	58/21 (73.4%)	<0.05^ab^
Diabetes mellitus, *n* (%)	67/42 (61.5%)	44/24 (64.7%)	60/19 (75.9%)	0.105
Hyperlipidemia, *n* (%)	85/24 (78%)	50/18 (73.5%)	62/17 (78.5%)	0.724
Daytime sleepiness, *n* (%)	79/30 (72.4%)	50/18 (73.5%)	28/51 (35.4%)	<0.05^b^
Snoring, *n* (%)	86/23 (78.8%)	45/23 (66.1%)	32/47 (40.5%)	<0.05^b^
STOP-Bang (scores)	4 (3,4)	3 (2,4)	3 (3,4)	<0.05^ab^
WBC (×10^9^/L)	9.04 ± 1.93	8.73 ± 2.03	8.88 ± 1.59	0.570
Neutrophil percentage	74.08 ± 4.74	67.42 ± 8.00	72.25 ± 4.71	<0.05^ab^
Lymphocyte percentage	17.41 ± 2.88	21.29 ± 5.91	19.49 ± 3.23	<0.05^ab^
NLR	4.36 ± 0.75	3.42 ± 0.97	3.65 ± 0.65	<0.05^ab^
PLR	132.48 ± 36.86	123.89 ± 28.95	126.41 ± 33.97	0.206
PLT (×10^9^/L)	199.33 ± 48.86	214.88 ± 42.86	212.06 ± 44.96	0.057
MPV (fl)	14.02 ± 2.29	13.52 ± 4.75	14.04 ± 3.10	0.541
MPV/PLT	7.46 ± 2.14	6.31 ± 1.87	6.82 ± 1.75	<0.05^a^
CRP (mg/L)	15.05 ± 4.27	14.94 ± 6.74	15.89 ± 5.96	0.897
D-dimer (ug/ml)	5.26 ± 1.94	5.63 ± 1.79	6.34 + 2.32	0.936

Notes: data were presented as mean ± standard deviation or median (25th and 75th percentiles) according to distribution normality. Normally distributed parameters of three groups were compared using one-way ANOVA test, whereas non-normally distributed parameters were compared using Kruskal–Wallis test, with Bonferroni adjustment for post hoc analysis. Categorical variables: Pearson's chi-square test was used for comparisons among three groups. ^a^Statistical significance between groups A and B. ^b^Statistical significance between groups A and C. BMI, body mass index; CRP, C-reactive protein; MPV, mean platelet volume; MPV/PLT, mean platelet volume to platelet ratio; NLR, neutrophil-to-lymphocyte ratio; PLR, platelet-to-lymphocyte ratio; PLT, platelet count.

**Table 3:** Comparison of sleep study parameters between three groups.

Variables	Group A	Group B	Group C	*P* value
TST (min)	303.13 ± 20.47	301.85 ± 25.00	301.06 ± 21.28	0.769
SE (%)	75.03 (73.22, 77.78)	74.67 (75.57, 76.46)	82.89 (81.69, 84.51)	<0.05^abc^
AHI (events/h)	43.86 ± 7.83	27.86 ± 4.12	2.49 ± 1.01	<0.05^ab^
MAI (events/h)	42.99 ± 4.07	43.08 ± 4.41	8.05 ± 2.24	<0.05^ab^
ODI (events/h)	52.58 ± 6.47	53.26 ± 6.19	5.17 ± 2.13	<0.05^ab^
LSpO_2_ (%)	71.50 ± 2.72	71.44 ± 2.66	95.54 ± 1.10	<0.05^ab^
*T* < 90% (%)	30.01 ± 2.09	31.36 ± 2.16	2.06 ± 0.84	<0.05^ab^

Notes: data were presented as mean ± standard deviation or median (25th and 75th percentiles) according to distribution normality. Normally distributed parameters of three groups were compared using one‐way ANOVA test, whereas non‐normally distributed parameters were compared using Kruskal–Wallis test, with Bonferroni adjustment for post hoc analysis. ^a^Statistical significance between groups A and C. ^b^Statistical significance between groups B and C. ^C^Statistical significance between groups A and B. AHI, apnea and hypopnea index; LSpO_2_, lowest oxygen saturation; MAI, microarousal index; ODI, oxygen desaturation index; TST, total sleep time; SE, sleep efficiency; *T*<90%, percentage of total sleep time spent with oxyhemoglobin saturation below 90%.

**Table 4:** Univariate and multivariate logistic regression analysis results: significant variables for predicting OSA in TBAD patients (groups A and C).

Variables	Univariate analysis			Multivariate analysis		
	OR	95% CI	*P* value	OR	95% CI	*P* value
BMI	0.96	0.84–1.10	0.58			
PLR	1.01	0.99–1.02	0.21			
STOP-Bang	1.80	1.31–2.43	<0.05^a^	1.97	1.34–2.90	<0.05^a^
NLR	3.61	2.27–5.74	<0.05^a^	3.73	2.27–6.15	<0.05^a^
MPV/PLT	1.17	1.03–1.36	<0.05^a^	1.16	0.97–1.39	0.08

Notes: the multivariate analysis was performed on variables with the *P* < 0.1 value. ^a^Statistical significance. AHI, apnea and hypopnea index; BMI, body mass index; MPV/PLT, mean platelet volume to platelet ratio; NLR, neutrophil to lymphocyte ratio; PLR, platelet to lymphocyte ratio.

**Table 5:** Diagnostic accuracy of NLR and STOP-Bang questionnaire in predicting OSA in AD patients (groups A and C) with the best cutoff value.

	AUC	95% CI	*P* value	Youden index	Cutoff value	Sensitivity	Specificity
NLR	0.75	0.68–0.82	<0.05	0.51	4.27	0.65	0.86
STOP-Bang	0.63	0.55–0.71	<0.05	0.16	3.50	0.59	0.57

Notes: AUC, area under ROC curve; NLR, neutrophil-to-lymphocyte ratio.

## Data Availability

The raw data required to reproduce these findings cannot be shared at this time as the data also form part of an ongoing study.

## Abbreviations

AD: Aortic dissection
AHI: Apnea and hypopnea index
AIMH: Aortic intramural hematoma
AUC: Area under ROC curve
BMI: Body mass index
CI: Confidence interval
CRP: C-reactive protein
LSpO_2_: The lowest oxygen saturation of peripheral blood
MAI: Microarousal index
MPV: Mean platelet volume
MPV/PLT: Mean platelet volume to platelet ratio
NLR: Neutrophil-to-lymphocyte ratio
NPV: Negative predictive value
ODI: 3% oxygen desaturation index
OR: Odds ratio
OSA: Obstructive sleep apnea
PLR: Platelet-to-lymphocyte ratio
PLT: Platelet count
PPV: Positive predictive value
PSG: Polysomnography
ROC: Receptor operating characteristic
SE: Sleep efficiency
SpO_2_: Oxygen saturation of peripheral blood
TAAD: Type A aortic dissection
TBAD: Type B aortic dissection
TB-AIMH: Type B aortic intramural hematoma
TST: Total sleep time
*T* < 90%: Percentage of total sleep time spent with oxyhemoglobin saturation below 90%
WBC: White blood cell count.
